# Supplementary material for: A risk stratification model for high-flow nasal cannula use in patients with coronavirus disease 2019 in Japan: A single-center retrospective observational cohort study
Source: PLoS One. 2024 Feb 23;19(2):e0290937. doi: 10.1371/journal.pone.0290937 (PMC10889628; doi:10.1371/journal.pone.0290937)
Supplement: Attachment — Submitted filename: Response to Reviewers.docx [file pone.0290937.s002.docx]

5. Review Comments to the Author

Reviewer #1: The authors present their single-center retrospective observational cohort study aiming to identify predictors of respiratory failure. I found this to be a simple and elegant study.

Major comments:

1. The definition of the primary outcome (respiratory failure) covers a wide spectrum of pathology from requiring more than 10L/ min oxygen to requiring mechanical intubation or death. I don't find this to be clinically useful. Is it possible for the authors to look at more meaningful clinical categories of outcomes (eg. requiring high flow oxygenation vs requiring non-invasive ventilation vs requiring invasive mechanical ventilation)?

→ Thank you for your comment. As per your comment, we changed the primary outcome from the critically respiratory illness (composite outcome, including oxygenation flow rate ≥10 L/min, high-flow oxygenation, noninvasive ventilation, invasive ventilation, and death) to require high-flow nasal cannula (HFNC), and redid our statistical analysis.

We revised manuscript and table as follows:

Line 25:

The primary outcome is the need for HFNC.

Line 30:

The rate of the need for HFNC was 22.9%.

Line 35-36:

The IBC-S score, an easy-to-use risk score based on parameters available at the time of hospital admission, predicted the need for HFNC in patients with COVID-19.

Line 66:

The primary outcome is the need for HFNC.

Line 68-69:

We excluded patients who needed oxygenation flow rate ≥10 L/min, needed HFNC and mechanical ventilation, or died on admission day.

Line 116-119:

Of these 152 patients, 4 patients who needed oxygenation flow rate ≥10 L/min, needed HFNC and mechanical ventilation, or died on admission day were excluded; therefore, the final analysis included 148 patients.

Line 121-122:

Fig 1 Study flow diagram

HFNC: high-flow nasal cannula; COVID-19: coronavirus disease 2019

Line 126-127:

Of the 148 patients, 34 patients (22.9%) required HFNC, 9 (6.1%) patients required invasive ventilation, and 13 patients (8.8%) died.

Table 1:

We added the clinical course (HFNC, mechanical ventilation and death).

Line 131-136：

A total of 19 variables that were measured at the time of admission were included in the least absolute shrinkage and selection operator regression, which identified SpO_2_ and IL-6 as significant predictors of the need for HFNC. In the logistic regression model including these two parameters, both an SpO2 <92% (odds ratio [OR] 7.50, 95% confidence interval [CI] 2.806–20.82; P < 0.001) and IL-6 (per 10 pg/ml increase, OR 1.23, 95% CI 1.010–1.038; P < 0.001) were independent and significant predictors of the need for HFNC and were included in the risk score (Table 2).

Table2:

Table 2 Association of SpO_2_ <92% and IL-6 with the risk of the need for HFNC

Line 138-140:

The cut-off IL-6 value of 19.5 pg/mL exhibited equal sensitivity and specificity with the highest positive predictive value of 63.2 pg/mL; therefore, this value was used to create the IL-6-based COVID-19 severity [IBC-S] score to determine the risk of the need for HFNC (Table 3).

Table3:

Table 3 IL-6 based COVID-19 severity score to predict the need for HFNC

Minor comments:

1. It is unclear to me how many primary care settings will have access to IL6 measurement.

→ Thank you for your comment. IL-6 can be measured at common testing facilities, and IL-6 measurement ELISA kits are available; thus, it can be measured in primary care settings as well.

We revised manuscript as follows:

Line 176-178:

IL-6 can be measured at common diagnostic facilities, and there are commercially available IL-6 measurement ELISA kits, enabling measurement in primary care settings.

2. In the introduction the authors should note that non-invasive ventilation/ high flow nasal cannula oxygenation may be injurious and result in patient self induced lung injury.

→ Thank you for your comment. We have added the following sentence:

Line 46-48:

In contrast, HFNC may promote further lung injury (e.g., self-inflicted lung injury) through vigorous breathing efforts.

Reviewer #2: In this manuscript, the authors determined the risk factors for HFNC use in patients with COVID-19 by examining the medical records of patients during the COVID-19 Alpha, Beta, and Delta surges in Japan. They found that among the 19 potential variables, SpO2 <92% (odds ratio [OR] 7.50, 95% confidence interval [CI] 2.806–20.82) and IL-6 (OR 1.021, 95% CI 1.010–1.033) were included in developing the risk score, which was termed IBC-S score. Although it is a well-written manuscript evaluating the severity of COIVD-19, I interpreted that several serious concerns existed.

1) Although they tried to determine the risk factors for needing HFNC support, they evaluated patients who needed oxygenation flow rate ≥10 L/min, high-flow oxygenation, noninvasive ventilation, invasive ventilation, and death. I do not think these patients are representative of patients that need HFNC.

→ Thank you for your comment. As per your comment, we changed the primary outcome from the critically respiratory illness (composite outcome, including oxygenation flow rate ≥10 L/min, high-flow oxygenation, noninvasive ventilation, invasive ventilation, and death) to require high-flow nasal cannula (HFNC), and redid our statistical analysis.

We revised manuscript and table as follows:

Line 25:

The primary outcome is the need for HFNC.

Line 30:

The rate of the need for HFNC was 22.9%.

Line 35-36:

The IBC-S score, an easy-to-use risk score based on parameters available at the time of hospital admission, predicted the need for HFNC in patients with COVID-19.

Line 66:

The primary outcome is the need for HFNC.

Line 68-69:

We excluded patients that needed oxygenation flow rate ≥10 L/min, needed HFNC, needed mechanical ventilation or died on admission day.

Line 116-119:

Of these 152 patients, 4 patients who needed oxygenation flow rate ≥10 L/min, needed HFNC, needed mechanical ventilation or died on admission day were excluded; therefore, the final analysis included 148 patients.

Line 121-122:

Fig 1 Study flow diagram

HFNC, high-flow nasal cannula; COVID-19, coronavirus disease 2019

Line 126-127:

Of the 148 patients, 34 patients (22.9%) required HFNC, 9 (6.1%) patients required invasive ventilation, and 13 patients (8.8%) died.

Table 1:

We added the clinical course (HFNC, mechanical ventilation and death).

Line 131-136：

A total of 19 variables that were measured at the time of admission were included in the least absolute shrinkage and selection operator regression, which identified SpO_2_ and IL-6 as significant predictors of the need for HFNC. In the logistic regression model including these two parameters, both an SpO2 <92% (odds ratio [OR] 7.50, 95% confidence interval [CI] 2.806–20.82; P < 0.001) and IL-6 (per 10 pg/ml increase, OR 1.23, 95% CI 1.010–1.038; P < 0.001) were independent and significant predictors of the need for HFNC and were included in the risk score (Table 2).

Table2:

Table 2 Association of SpO_2_ <92% and IL-6 with the risk of the need for HFNC

Line 138-140:

The cut-off IL-6 value of 19.5 pg/mL exhibited equal sensitivity and specificity with the highest positive predictive value of 63.2 pg/mL; therefore, this value was used to create the IL-6-based COVID-19 severity [IBC-S] score to determine the risk of the need for HFNC (Table 3).

Table3:

Table 3 IL-6 based COVID-19 severity score to predict the need for HFNC

2) They included measurement of IL-6 in their score. I do not think use of HFNC is limited to special hospitals, because it is already used at home. When the patient is in a hospital where they can measure IL-6, I guess they can use HFNC.

→ Thank you for your comment. As mentioned by you, use of HFNC is not limited to special hospitals, because it is already used at home.

Thus, we deleted the sentence.

Line 51 before revise

However, HFNC uses hyperbaric oxygen, which limits the medical facilities that can utilize it.
