# Supplementary material for: A risk stratification model for high-flow nasal cannula use in patients with coronavirus disease 2019 in Japan: A single-center retrospective observational cohort study
Source: PLoS One. 2024 Feb 23;19(2):e0290937. doi: 10.1371/journal.pone.0290937 (PMC10889628; doi:10.1371/journal.pone.0290937)
Supplement: Attachment — Submitted filename: Response to Reviewer 2.docx [file pone.0290937.s003.docx]

5. Review Comments to the Author

Reviewer #1: The authors have responded to all of the comments. The manuscript makes a helpful contribution to the literature.

→ Thank you for your valuable comment.

Reviewer #2: According to the suggestions by the reviewer, the authors changed their primary outcome from the critically respiratory illness (composite outcome, including oxygenation flow rate ≥10 L/min, high-flow oxygenation, noninvasive ventilation, invasive ventilation, and death) to require high-flow nasal cannula (HFNC), and redid their statistical analysis.

Major comments

1) It looks strange to me that their non-outcome group contains patients that did not require HFNC and 9 patients that required invasive ventilation, and 13 patients that died. How is the disease severity of the non-outcome group?

→ Thank you for your insightful comment.

Table 1 shows that “non-outcome group (n = 114)” comprises no patients who required HFNC, invasive ventilation, or died, and that “outcome group (n = 34)” consisted of 34 patients who required HNFC for the primary outcome, of which 9 patients required invasive ventilation and 12 patients died.

2) If the introduction of HFNC is the primary outcome, please explain the introducing criteria of HFNC at your institution.

→ Thank you for your valuable comment. Accordingly, we have added the sentence and two references for explaining the criteria for introduction of HFNC at our institution as follows;

Page 4, Line 66-69

HFNC was administered to patients who were unable to maintain an SpO_2_ level of above 92% and respiratory rate below 25 breaths per minute, even when they received standard oxygen administered through a face mask at the flow rate of 10 L/min or higher [8,9].

8.	Xu J, Yang X, Huang C, Zou X, Zhou T, Pan S, et al. A novel risk-stratification models of the high-flow nasal cannula therapy in COVID-19 patients with hypoxemic respiratory failure. Front Med (Lausanne). 2020;7:607821. doi: 10.3389/fmed.2020.607821, PMID 33425951.

9.	Roca O, Caralt B, Messika J, Samper M, Sztrymf B, Hernández G, et al. An index combining respiratory rate and oxygenation to predict outcome of nasal high-flow therapy. Am J Respir Crit Care Med. 2019;199(11):1368-76. doi: 10.1164/rccm.201803-0589OC, PMID 30576221.

Minor comments

Please explain what "outcome" and "non-outcome" mean in the Tables and Figures.

→ Thank you for your valuable comment. Accordingly, we have marked "outcome" in Table 1 with one asterisk and “non-outcome” with two asterisks, and described the meaning of each in the footnotes as follows. Additionally, we have added the sentence in Fig 1 caption as follows:

Table1:

*Indicates requirement of HFNC, and **Indicates no requirement of HFNC.

Fig 1 **Study flow diagram.** Outcome indicates requirement of HFNC, Non-Outcome indicates no requirement of HFNC.

HFNC: high-flow nasal cannula; COVID-19: coronavirus disease 2019
